# Gibberellin Application at Pre-Bloom in Grapevines Down-Regulates the Expressions of *VvIAA9* and *VvARF7*, Negative Regulators of Fruit Set Initiation, during Parthenocarpic Fruit Development

**DOI:** 10.1371/journal.pone.0095634

**Published:** 2014-04-17

**Authors:** Chan Jin Jung, Youn Young Hur, Hee-Ju Yu, Jung-Ho Noh, Kyo-Sun Park, Hee Jae Lee

**Affiliations:** 1 Fruit Research Division, National Institute of Horticultural and Herbal Science, Rural Development Administration, Suwon, Republic of Korea; 2 Department of Plant Science, Seoul National University, Seoul, Republic of Korea; 3 Department of Life Sciences, The Catholic University of Korea, Bucheon, Republic of Korea; 4 Research Institute for Agriculture and Life Sciences, Seoul National University, Seoul, Republic of Korea; Instituto de Biología Molecular y Celular de Plantas, Spain

## Abstract

Fruit set is initiated only after fertilization and is tightly regulated primarily by gibberellins (GAs) and auxins. The application of either of these hormones induces parthenocarpy, fruit set without fertilization, but the molecular mechanism underlying this induction is poorly understood. In the present study, we have shown that the parthenocarpic fruits induced by GA application at pre-bloom result from the interaction of GA with auxin signaling. The transcriptional levels of the putative negative regulators of fruit set initiation, including *Vitis auxin/indole-3-acetic acid transcription factor 9* (*VvIAA9*), *Vitis auxin response factor 7* (*VvARF7*), and *VvARF8* were monitored during inflorescence development in seeded diploid ‘Tamnara’ grapevines with or without GA application. Without GA application, *VvIAA9, VvARF7*, and *VvARF8* were expressed at a relatively high level before full bloom, but decreased thereafter following pollination. After GA application at 14 days before full bloom (DBF); however, the expression levels of *VvIAA9* and *VvARF7* declined at 5 DBF prior to pollination. The effects of GA application on auxin levels or auxin signaling were also analyzed by monitoring the expression patterns of auxin biosynthesis genes and auxin-responsive genes with or without GA application. Transcription levels of the auxin biosynthesis genes *Vitis anthranilate synthase β subunit* (*VvASB1-like*), *Vitis YUCCA2* (*VvYUC2*), and *VvYUC6* were not significantly changed by GA application. However, the expressions of *Vitis Gretchen Hagen3.2* (*VvGH3.2*) and *VvGH3.3*, auxin-responsive genes, were up-regulated from 2 DBF to full bloom with GA application. Furthermore, the *Vitis* GA signaling gene, *VvDELLA* was up-regulated by GA application during 12 DBF to 7 DBF, prior to down-regulation of *VvIAA9* and *VvARF7*. These results suggest that *VvIAA9* and *VvARF7* are negative regulators of fruit set initiation in grapevines, and GA signaling is integrated with auxin signaling via *VvDELLA* during parthenocarpic fruit development in grapevines.

## Introduction

Fruit set is initiated only after two sequential events, pollination and fertilization [1], concurrent with changes in the levels of endogenous plant hormones, primarily gibberellins (GAs) and auxins [2]–[5]. Application of GA or auxin can trigger fruit set even without pollination and can induce parthenocarpic fruit development [6]–[10].

Although the molecular mechanisms by which either GA or auxin mediates fruit set initiation are not clearly established, several auxin signaling genes related to parthenocarpic fruit development have been identified. The auxin/indole-3-acetic acid (Aux/IAA) transcription factor family is a known essential repressor of auxin signaling in various developmental processes, including fruit set [11]. Among the Aux/IAA (IAA) family genes, *IAA9* has been regarded as a negative regulator, preventing fruit set initiation in the absence of pollination in tomato, with the silencing line, *SlIAA9*, showing parthenocarpic fruit development [12]. Two auxin response factors (ARF) related to parthenocarpic fruit development have also been identified in *Arabidopsis* and tomato [12]–[15]. The ARF family acts as a regulator of auxin-responsive genes by specifically binding to auxin response elements (AuxREs) in the promoters of auxin-responsive genes. ARFs play important roles in diverse developmental processes in embryos, hypocotyls, floral organs, and fruit [15]–[21]. The *Arabidopsis fruit without fertilization* (*fwf*) mutant produces parthenocarpic fruit as the result of the expression of truncated *ARF8* [14], [15], and the same mutation in the *SlARF8* in tomato induced parthenocarpy [14]. Parthenocarpy induction and pollen tube growth inhibition were also observed in a silencing line of *ARF7* in tomato [13], [22]. The expression of *SlARF7* was maintained at high levels before pollination and rapidly declined after pollination with increasing auxin content [1]–[3], [6], in a manner similar to its down-regulation upon exogenous auxin application [13].

GA-induced parthenocarpic fruit development has been observed in tomato mutants (*pat*, *pat-2*, and *pat-3/pat-*4) showing overexpression of the GA biosynthesis genes [23]–[25], and in silencing lines of *DELLA* in *Arabidopsis* [26] and tomato [27], demonstrating that GA signaling plays a role in parthenocarpic fruit development. The regulatory roles of the DELLA protein in *ARF7* expression, and the partial activation of auxin signaling during parthenocarpic fruit development have been reported in the *procera* (*pro*) mutant of *DELLA* in tomato [28]. Additionally, auxin application and the silencing line of *ARF7* induced parthenocarpy by regulating expression levels of the GA metabolic genes [22], [29], [30]. These data suggest that both GA and auxin influence fruit set initiation, and crosstalk between GA and auxin signaling plays a role in parthenocarpic fruit development.

In grapevines, GA has commonly been used to induce parthenocarpy [2], [7], [8], [31]. The effects of GA application have been studied on early ripening and berry enlargement [32]–[34], and on the appropriate application timing at the pre-bloom stage for the induction of seedless grapes [31], [35]. On a molecular level, both GA and auxin biosynthesis genes are up-regulated after pollination in grapevines [3], [36], [37]. However, although GA-mediated parthenocarpy is a highly desirable trait for table grapes, how GA induces parthenocarpic fruit development remains unclear. GA application at the pre-bloom stage in grapevines inhibited pollen tube growth and disturbed the balance of GA metabolism at near full bloom [37]. In the present study, changes in the expression levels of the fruit set related genes, *VvIAA9*, *VvARF7*, *VvARF8*, and *VvDELLA* in grapevines were monitored to determine whether GA application coordinates auxin signaling during parthenocarpic fruit development.

## Materials and Methods

### Plant Material and GA Application

Five-year-old grapevines of the seeded diploid cultivar ‘Tamnara’ (*Vitis* spp.), grown in an overhead arbor system, were used for the GA application and gene expression analysis. The cultivar used in this study was bred from a cross between ‘Campbell Early’ (*V. labruscana*) and ‘Himrod’ (*Vitis* spp.) at the National Institute of Horticultural and Herbal Science, Suwon, Republic of Korea in 1998 [38]. A GA solution (Dongbu, Seoul, Korea) at 100 ppm was applied as described by Okamoto and Miura [35] onto inflorescence clusters 14 days before full bloom (DBF), which corresponded to the stage showing eight separated leaves and a compact grouped flower, based on the E-L system of Coombe [39], and they were labeled. Clusters were harvested at 0, 1, 2, 4, 7, 9, 12, 14, 16, and 19 days after GA application. Harvested inflorescence samples were immediately frozen in liquid N_2_, and stored at −80°C until RNA extraction.

### Protein Identification

The *Vitis* homologous proteins ARF7, ARF8, YUCCA2 (YUC2), YUC6, and DELLA were identified using a BLASTP search, except for the previously identified *Vitis* IAA9 (VvIAA9) (HQ337788) [40] and VvGAI1 (XP_002284648) [41], [42]. Amino acid sequences for the *Arabidopsis* ARF, Aux/IAA, YUC, and DELLA family proteins were obtained from the National Center for Biotechnology Information (NCBI). For tomato, however, amino acid sequences of the homologues were acquired from previous genome-wide studies [43]–[45]. Using these sequences, iTAK (http://bioinfo.bti.cornell.edu/cgi-bin/itak/index.cgi), the Plant Transcription Factor Database, version 3.0 (PlnTFDB, http://plntfdb.bio.uni-potsdam.de/v3.0/), and GreenPhyl (http://www.greenphyl.org/cgi-bin/index.cgi) were screened, and protein sequences were confirmed using the NCBI. The gene sequences of *VvARF7* (GSVIVT01015035001), *VvARF8* (GSVIVT01035204001), *VvYUC2* (GSVIVT01015388001), *VvYUC6* (GSVIVT01035678001), and *VvDELLA* (GSVIVT01030735001) were identified using the Grape Genome Browser, version 12X (http://www.genoscope.cns.fr/externe/GenomeBrowser/Vitis/). Distinct domains for each protein were predicted using PROSITE (http://prosite.expasy.org/prosite.html), Pfam (http://pfam.janelia.org/), and previous reports of ARFs [13], [46], [47], YUCs [48], [49], and DELLAs [50]. Accession numbers for all amino acid sequences used in this study are listed in Table S1.

### Phylogenetic Analysis

Protein sequence alignments were generated using ClustalW version 2.1 for multiple alignments, and phylogenetic analyses were performed using neighbor-joining algorithms of the MEGA5 program [51] with the pairwise-deletion option. One thousand replicates were used in the bootstrap analysis.

### Total RNA Isolation and cDNA Synthesis

Total RNA was isolated from whole inflorescence samples including berries and pedicels, at various developmental stages according to an RNA extraction method [52] modified to remove polysaccharides and phenolic compounds. The cDNA was synthesized by reverse transcription of 0.5 µg RNA using the PrimeScript first-strand cDNA Synthesis Kit (Takara, Tokyo, Japan) with an oligo-dT primer, according to the manufacturer’s instructions.

### Quantitative Reverse Transcription-polymerase Chain Reaction (qRT-PCR)

cDNA was subjected to qRT-PCR using the gene-specific forward and reverse primers shown in Table S2. Primers used in previous studies [36], [53] were used for the grapevine auxin biosynthesis gene, the putative *Vitis* anthranilate synthase β subunit-like (*VvASB1-like*), and two auxin signaling *Vitis Gretchen Hagen3* (*GH3*) family genes. qRT-PCR was performed with the SYBR Premix Ex Taq (Takara) on a Thermal Cycler Dice Real-Time System TP800, version 4.0 (Takara), under universal thermal cycling conditions described by the manufacturer. *Vitis Actin1* was used as an endogenous control for normalization of gene expression. With cycle threshold (C_T_) values obtained from the qRT-PCR results, the ΔC_T_ value (C_T target gene_ C_T *VvActin1*_) was calculated for each gene. The relative expression of each gene normalized to the ΔC_T_ value of samples from 14 DBF was determined using the comparative C_T_ method (2^ΔΔCT^). Analysis of qRT-PCR efficiency showed that all amplicons of all genes used in this study were in the optimal range of 95–105% (Figure S1).

## Results

### Characterization and Identification of *Vitis* ARF7 and ARF8

**Figure 1:**
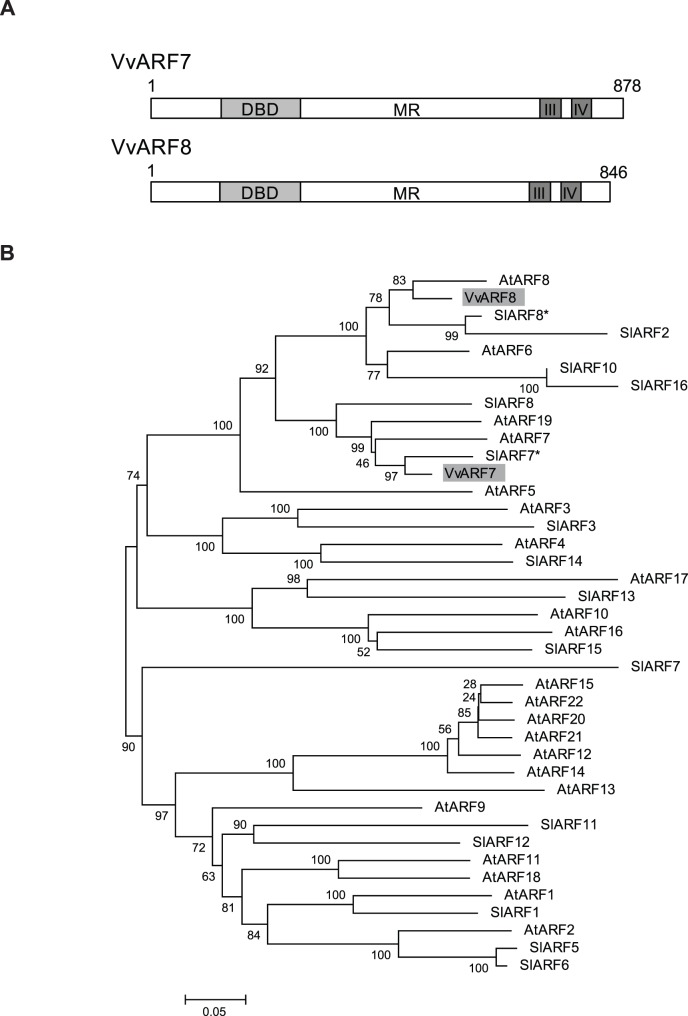
Protein sequence alignment and phylogenetic analysis of VvARF7 and VvARF8. (**A**) Schematic diagrams of VvARF7 and VvARF8. The length of each protein is denoted. DBD, B3 DNA-binding domain; MR, middle region; III, Aux/IAA dimerization domain III; IV, Aux/IAA dimerization domain IV. (**B**) Phylogenetic tree of *Vitis* ARF7 and ARF8 with *Arabidopsis* and tomato ARF families. Asterisks indicate previously reported SlARF7 and SlARF8 proteins, numbered SlARF9 and SlARF4 [46], respectively, based on their locations on the chromosome.

Two ARF family proteins, VvARF7 and VvARF8, were identified from several *Vitis* transcription factor databases. VvARF7 and VvARF8 were previously reported as VvIAA24 and VvIAA7, respectively [54]. Using several plant transcription databases and PROSITE and Pfam, the VvARF7 amino acid sequence was deduced to contain an N-terminal B3-type DNA-binding domain (DBD; amino acids 126–228), a middle region (MR), two C-terminal Aux/IAA dimerization domains, domain III (amino acids 753–788), and domain IV (amino acids 797–839), needed for typical ARF protein activities. The VvARF8 sequence was also deduced to contain an N-terminal DBD (amino acids 128–230), a MR, two C-terminal Aux/IAA dimerization domains, domain III (amino acids 721–756), and domain IV (amino acids 764–807) (Figure 1A). Furthermore, the VvARF7 sequence showed 63 and 61% identity with AtARF7 and SlARF7, respectively, and VvARF8 had 68% identity with AtARF8 and more than 71% identity with SlARF8 (Figure S2). Thus, we renamed these proteins VvARF7 and VvARF8 to comply with the nomenclature of the *Arabidopsis* ARFs, based on the similarity between these proteins. Phylogenetic analysis and comparison of VvARF7 and VvARF8 with *Arabidopsis* and tomato ARFs showed that both proteins were clustered with the transcription-activating AtARFs, AtARF5, 6, 7, 8, and 19, according to Guilfoyle and Hagen [55] (Figure 1B).

### Down-regulation of *VvIAA9* and *VvARF7* with GA Application

**Figure 2:**
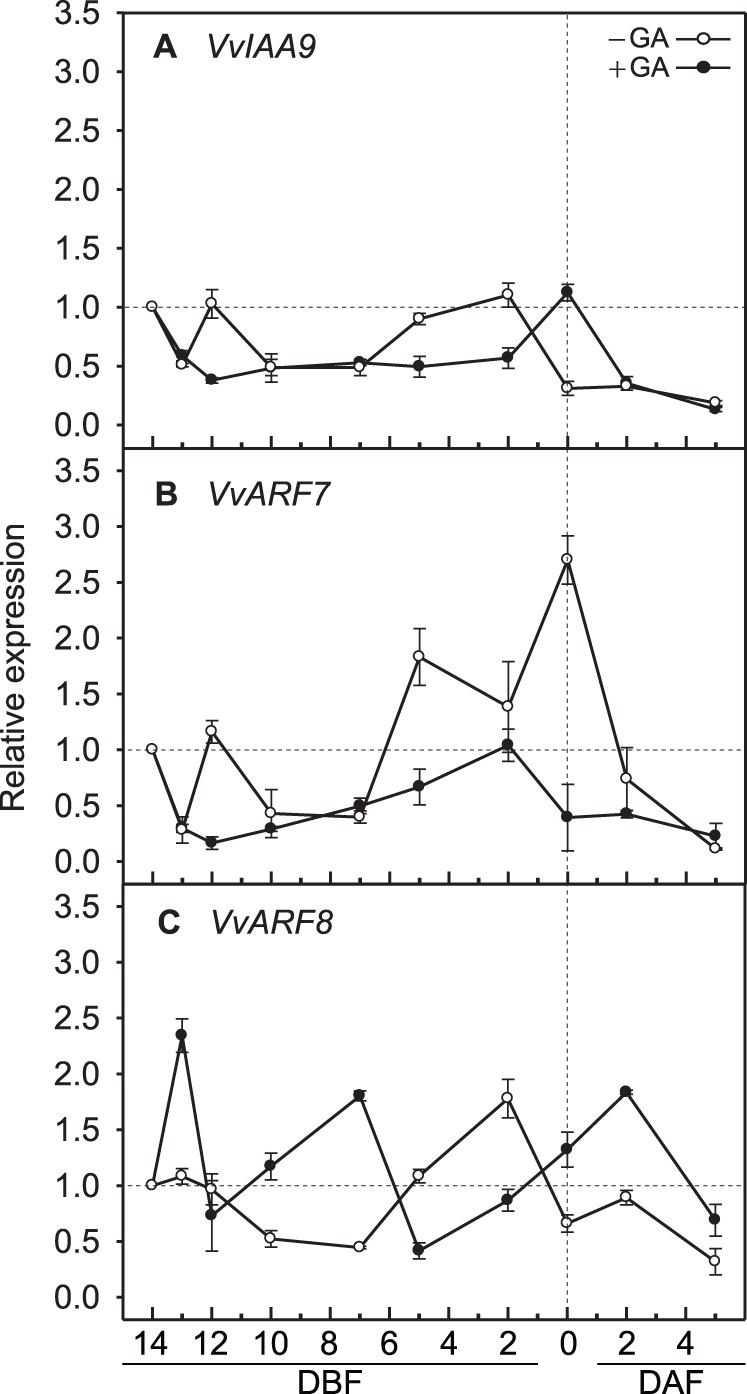
Transcriptional changes in *Vitis* negative regulator genes for fruit set initiation. (**A**) *VvIAA9*, (**B**) *VvARF7*, and (**C**) *VvARF8* in grapevine inflorescences with and without GA application. DAF, days after full bloom; DBF, days before full bloom. Quantitative reverse transcription-PCR was performed to measure the expression level of each gene using the comparative C_T_ method. After normalization of each sample to the expression of the internal control *VvActin1*, the expression of each gene relative to the 14 DBF sample was calculated. Bars are standard errors of the means from three independent experiments.

Using GA applied inflorescence clusters at 14 DBF, which induces parthenocarpy in the seeded diploid ‘Tamnara’ grapevines, relative transcription levels of *VvIAA9*, *VvARF7*, and *VvARF8* during inflorescence development were analyzed to determine whether GA-induced parthenocarpy in grapevines is associated with auxin-related genes. Without GA application, the expression patterns of *VvIAA9* and *VvARF7* changed similarly until 7 DBF, except for peak expression times of *VvIAA9* and *VvARF7* at 2 DBF and full bloom, respectively, following which the expression levels of both genes rapidly declined (Figure 2A–B). *VvARF8* also showed the highest expression at 2 DBF, with a rapid down-regulation without GA application. With GA application, *VvIAA9* and *VvARF7* expressions at 12 DBF remained at approximately 50 and 14% of the levels observed without GA application, respectively. Furthermore, the expression levels of *VvIAA9* at 2 DBF and *VvARF7* at 5 DBF were significantly lower, dropping to 50 and 37% compared to without GA application, respectively, and *VvARF7* expression further reduced to 15% at full bloom, compared to without GA application (Figure 2A–B). However, *VvARF8* had a fluctuating expression pattern with GA application, showing a more than 2-fold up-regulation from 14 DBF to 5 DAF, and a down-regulation over 20 and 50% at 12 DBF, and during 5 to 2 DBF, respectively, compared to without GA application (Figure 2C). These results indicate that *VvIAA9*, *VvARF7*, and *VvARF8* expressions were maintained at high levels before pollination and rapidly declined after pollination, but GA application at pre-bloom, down-regulated *VvIAA9* and *VvARF7* without pollination.

### Up-regulation of *VvGH3.2* and *VvGH3.3* with GA Application

**Figure 3:**
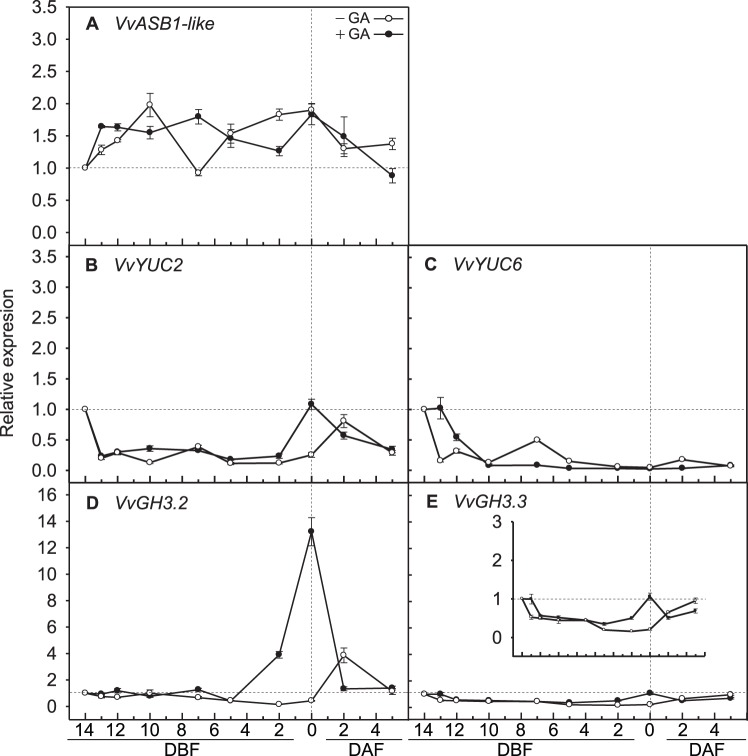
Transcriptional changes in auxin biosynthesis genes (A, B, and C) and early auxin-responsive genes (D and E). (**A**) *VvASB1-like*, (**B**) *VvYUC2*, (**C**) *VvYUC6*, (**D**) *VvGH3.2*, and (**E**) *VvGH3.3* in grapevine inflorescences with and without GA application. The expression level of *VvGH3.3* is magnified in the insets. DAF, days after full bloom; DBF, days before full bloom. Quantitative reverse transcription-PCR was performed to measure the expression level of each gene using the comparative C_T_ method. After normalization of each sample to the expression of the internal control *VvActin1*, the expression of each gene relative to the 14 DBF sample was calculated. Bars are standard errors of the means from three independent experiments.

To determine whether auxin or auxin signaling was affected by GA application in grapevines, the expression patterns of auxin biosynthesis and the auxin-responsive genes were analyzed during inflorescence development. Auxin biosynthesis was verified by analyzing the expression patterns of the *VvASB1-like*, *VvYUC2*, and *VvYUC6* genes, which encode key enzymes in auxin biosynthesis. The expression of *VvASB1-like* was reported by Dauelsberg et al. [36], and *VvYUC2* and *VvYUC6* were found to be the closest *Vitis* homologue genes of *Arabidopsis YUC2* (*AtYUC2*) and *AtYUC6*, and the tomato *YUC2* homologue, *ToFZY2*, which were abundantly expressed in flowers [43], [48]. The VvYUC2 and VvYUC6 proteins shared more than 65% identity and had highly conserved FAD and NADPH binding sites with those of *Arabidopsis* and tomato (Figure S3). The levels of *VvASB1-like* transcript fluctuated and peaked at 10 DBF and full bloom, but both *VvYUC2* and *VvYUC6* were expressed at relatively low levels during inflorescence development without GA application, except for *VvYUC2*, which was up-regulated at 2 DAF (Figure 3A–C). With GA application, expression patterns of these auxin biosynthesis genes were not significantly different from those without GA application, except for the up-regulations of *VvASB1-like*, *VvYUC2*, and *VvYUC6* at 7 DBF, full bloom, and 13 DBF, respectively (Figure 3A–C). These results indicate that auxin biosynthesis was not affected by GA application. However, transcription levels of the early auxin-responsive genes were significantly changed following GA application. Without GA application, the expression of *VvGH3.2* and *VvGH3.3* declined steadily until 2 DBF and then increased after full bloom, with higher expression of *VvGH3.2* than *VvGH3.3*. With GA application, however, *VvGH3.2* and *VvGH3.3* showed significantly higher levels of transcription at full bloom, with increases of more than 26- and 5-fold, respectively (Figure 3D–E), suggesting that GA application altered auxin signaling.

### Up-regulation of *VvDELLA* with GA Application

**Figure 4:**
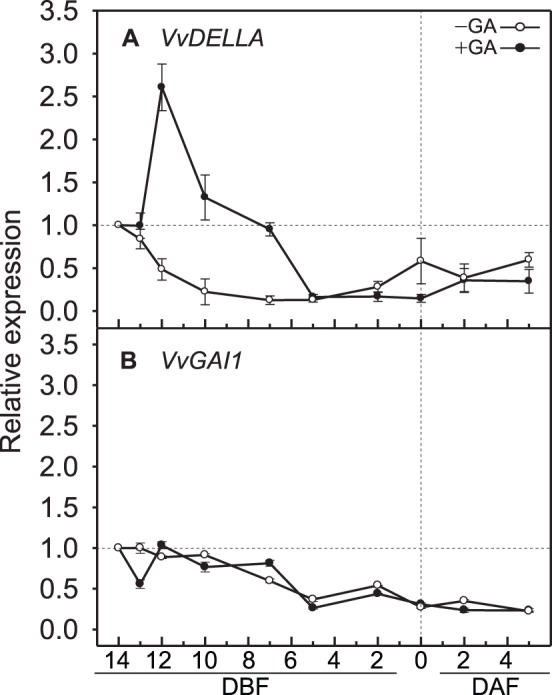
Transcriptional changes in two *DELLA* genes. (**A**) *VvDELLA* and (**B**) *VvGAI1* in grapevine inflorescences with and without GA application. DAF, days after full bloom; DBF, days before full bloom. Quantitative reverse transcription-PCR was performed to measure the expression level of each gene using the comparative C_T_ method. After normalization of each sample to the expression of the internal control *VvActin1*, the expression of each gene relative to the 14 DBF sample was calculated. Bars are standard errors of the means from three independent experiments.

The GA signal was analyzed by monitoring changes in the expression pattern of *VvDELLA* and *VvGAI1*, *Vitis* DELLA family genes, known as key integrators of GA and other hormonal signaling pathways [41], [56]. *VvDELLA* shares 64 and 66% identity with AtGAI and SlDELLA, respectively, and contains highly conserved functional motifs, such as DELLA, TVHYNP, and GRAS domains (Figure S4A–B). Without GA application, transcription levels of *VvDELLA* remained low throughout inflorescence development, and the expression levels of *VvGAI1* were continuously reduced (Figure 4). These expression patterns of *VvGAI1* were not affected by GA application (Figure 4B), but *VvDELLA* showed different expression patterns. One day after GA application, at 13 DBF, the expression level of *VvDELLA* was not significantly different from that observed without GA application. However, expression in *VvDELLA* increased more than 6-fold, from 12 to 7 DBF on GA application, and declined rapidly thereafter (Figure 4A).

## Discussion

### GA Application Induces Parthenocarpy by Early Down-regulation of Negative Regulators of Fruit Set Initiation

Parthenocarpic fruit development in mutants of the *IAA9*, *ARF7*, and *ARF8* genes was the result of reduced expression of these genes before pollination [5], [13]–[15]. Without GA application, *VvIAA9*, *VvARF7*, and *VvARF8* were highly expressed until near full bloom, after which their expressions declined after 2 DBF or full bloom (Figure 2). These results imply that *VvIAA9*, *VvARF7*, and *VvARF8* function as negative regulators of fruit set initiation, whose down-regulation leads to fruit set initiation. With GA application, however, *VvIAA9* and *VvARF7* were down-regulated 2 days after the application, and their transcription levels remained low until full bloom or 2 DBF, respectively (Figure 2A–B), in which expression levels of GA biosynthesis genes were down-regulated by GA application at 14 DBF [37]. These results indicate that effects of GA application at pre-bloom were not limited on transcriptional changes of these genes at 1 or 2 days after the application. An enlarged ovary and inhibition of pollen tube growth were observed, both in the pistil of grapevines with GA applied at 14 DBF [35], [37], and in *ARF7*-silencing lines [13], substantiating the down-regulatory effects of GA application on *VvARF7* expression at full bloom. These results also suggest that GA can substitute for the effects of auxin in fruit set initiation in the absence of pollination. Although *ARF8* has been reported as a negative regulator of fruit set initiation in *Arabidopsis* and tomato [14], [15], only truncated, and not null mutants of *ARF8* showed parthenocarpic fruit initiation [15], and direct involvement of *ARF8* in parthenocarpic fruit development was not observed in the present study.

To verify whether reduced transcription of *VvIAA9* and *VvARF7* with GA application was due to an increase in auxin levels, transcriptional changes were monitored in the auxin biosynthesis genes, *VvASB1-like*, *VvYUC2*, and *VvYUC6*, and two early auxin-responsive gene, *VvGH3.2* and *VvGH3.3*. *VvASB1-like* encodes the first step enzyme of auxin biosynthesis [57], and two *Vitis YUC* genes; *VvYUC2* and *VvYUC6* genes, encode the final step enzymes [58]. An accumulation of auxin, resulting from the up-regulation of these genes, has been reported in *Arabidopsis* [49], [57]–[59]. The up-regulation of *VvASB1-like* upon pollination has also been reported in grapevines [36]. Furthermore, auxin or auxin signaling mediated induction of the *GH3* gene family, which encode the early auxin-responsive IAA-amino synthetases, have been reported in *Arabidopsis* [60], [61] and tomato [13], [22]. In grapevines, six *Vitis GH3* family genes have been identified, and both *VvGH3.2*, the most abundantly expressed *Vitis GH3* gene in flower and *VvGH3.3*, the closest homolog of *AtGH3.6* in *Vitis* [53], showed auxin-inducible expression patterns. With GA application, there was only a slight fluctuation of the expression patterns of *VvASB1-like*, *VvYUC2*, and *VvYUC6* (Figure 3A–C), however *VvGH3.2* and *VvGH3.3* were up-regulated more than 3-fold between 2 DBF and full bloom (Figure 3D–E), when *VvIAA9* and *VvARF7* were down-regulated. With GA application, *VvGH3.2* was expressed 4-fold higher at full bloom, compared to without GA application at 2 DAF (Figure 3D), indicating that the up-regulation of *VvGH3.2* at full bloom was due to the GA application rather than to ovary development. These results are in agreement with the observed up-regulation of the *SlGH3-like* gene expression in a silencing line of *SlARF7* [13], and also support the idea that GA application down-regulates *VvARF7*, with partial activation of auxin signaling during parthenocarpic fruit development. However, the possibility of GA-mediated accumulation of auxin could not be excluded, since the peak expression time of *VvYUC2* correlated with the highest expression of *VvGH3.2* at full bloom with GA application (Figure 3B, D).

### Integration of GA and Auxin Signaling during Parthenocarpic Fruit Development via *VvDELLA*

The crosstalk between GA and auxin in fruit set initiation has been demonstrated by the effects of auxin on GA biosynthesis in *Arabidopsis* and tomato [29], [30], and the partial involvement of GA signaling in silencing lines of *ARF7* in tomato [13], [22]. Using the *pro* tomato mutant, a loss-of-function mutant of *SlDELLA*, Carrera et al. [28] demonstrated that GA affected a component of auxin signaling by down-regulating the negative regulators of fruit set initiation; *SlIAA9* and *SlARF7*. Thus, GA and auxin may activate each other in the signaling pathway to a certain extent, and this integration appears to be associated with parthenocarpy induction. The activation of auxin signaling by GA application observed in this study was consistent with the partial auxin signaling activation in silencing lines of *SlARF7* [13]. Furthermore, with GA application, the prior up-regulation of *VvDELLA* decreased of expression levels of *VvIAA9* and *VvARF7*, suggesting that GA application induced parthenocarpic fruits by reducing *VvIAA9* and *VvARF7* via *VvDELLA* (Figures 2A–B; 4A). The up-regulation of *VvDELLA* indicated that its transcription was under a GA-mediated feedback regulation, as observed in *SlDELLA* [28]. However, *VvGAI1*, another *Vitis* DELLA family gene, identified previously as a floral induction related *Vitis* DELLA family gene [41], did not show transcriptional changes, regardless of GA application, as did the *Arabidopsis* DELLA family genes. These differential transcriptional regulations by GA application between *VvDELLA* and *VvGAI1* may have originated from the differences in the amino acid sequences of the Poly S/T/N motif, the transcriptional regulatory region of DELLA proteins [56], [62], [63]. A comparison of the amino acid sequences of the Poly S/T/N motif showed that VvDELLA and SlDELLA shared more conserved amino acids than VvGAI1 and the amino acid sequences of VvGAI1 in this motif were similar to those of AtGAI and AtRGA (Figure S4C–D).

**Figure 5:**
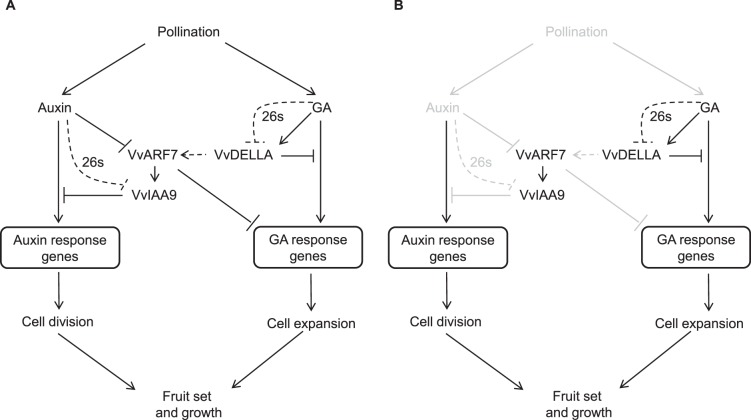
Proposed model for GA and auxin crosstalk in grapevines during fruit set initiation. The possible functions of *VvARF7*, *VvIAA9*, and *VvDELLA* are also included, based on de Jong et al. [6] and Carrera et al. [28]. (**A**) Pollination-mediated fruit set initiation. The dashed line with 26S indicates the regulation of protein levels by 26 s proteasome-mediated degradation, and the arrow indicates activation at the protein level. (**B**) Putative hormone signaling in fruit set initiation by GA application on grapevine inflorescences. Pathways inactivated by GA application are indicated in gray.

Based on the results presented in this study and previous data from de Jong et al. [22] and Carrera et al. [28], we propose a model for fruit set initiation that is mediated by *VvARF7*, *VvIAA9*, and *VvDELLA* in grapevines with or without GA application. Elevated GA and auxin upon pollination initiate fruit set by down-regulating *VvIAA9* and *VvARF7*, negative regulators of fruit set initiation (Figure 5A). With GA application, however, the consecutive transcriptional changes in *VvDELLA*, *VvIAA9*, and *VvARF7*; i.e., the up-regulation of *VvDELLA* followed by the down-regulation of *VvIAA9* and *VvARF7* during inflorescence development (Figures 2A–B; 4D), and the early reduction of *VvIAA9* and *VvARF7* may replace the effects of auxin and initiate parthenocarpic fruit development (Figure 5B).

In this study, we reviewed the complex expression patterns of fruit set initiation related genes in grapevines and have shown that GA application at pre-bloom down-regulates *VvIAA9* and *VvARF7* before pollination and activates some auxin signaling via *VvDELLA* during parthenocarpic fruit development. This is the first report to detail the molecular mechanism of fruit set initiation in grapevines, and contributes to improving fruit productivity by providing information to increase fruit set initiation of other important crops. The integration of GA and auxin signaling, including the roles of *VvIAA9*, *VvARF7*, and *VvDELLA* should be further investigated to broaden our understanding of the molecular mechanisms underlying GA-mediated induction of parthenocarpic fruit development in viticulture.

## Supporting Information

Figure S1 **qRT-PCR efficiency plots for GA metabolic genes.** Mean quantification cycle (C_T_) values obtained from 10-fold serial dilution series of each gene plotted against the logarithm of the cDNA template concentration. The amplification efficiency (E) was calculated by E = [10^(−1/S)^ −1]×100, where S = the slope of the linear regression line.
(PDF)

Figure S2 **Protein alignment and phylogenetic analysis of VvARF7 and VvARF8.** (**A**) Comparison of the AtARF7, SlARF7, and VvARF7 amino acid sequences. (**B**) Comparison of the AtARF8, SlARF8, and VvARF8 amino acid sequences. The B3 DNA-binding domain is denoted with an open box. The Aux/IAA dimerization domains III and IV are underlined with solid and dashed lines, respectively. Identical and similar amino acids are shaded in black and gray, respectively.
(PDF)

Figure S3 **Protein alignment and phylogenetic analysis of VvYUC2 and VvYUC6.** (**A**) Comparison of the AtYUC2, AtYUC6, ToFZY2, VvYUC2, and VvYUC6 amino acid sequences. The FAD and NADPH binding sites are underlined. Identical and similar amino acids are shaded in black and gray, respectively. (**B**) A phylogenetic tree comparing VvYUC2 and VvYUC6 with *Arabidopsis* and tomato YUCCA families.
(PDF)

Figure S4 **Protein sequence alignment and phylogenetic analysis of VvDELLA.** (**A**) Comparison of the AtGAI, SlDELL, and VvDELLA amino acid sequences. The DELLA and TVHYNP motifs for GA signal perception, the poly S/T/N motif for regulation of DELLA expression and the NLS motif for nuclear localization are denoted with open boxes. The LHR and VHIID domains for dimerization of DELLA protein are indicated with solid and dashed lines, respectively. The PFYRE and SAW domains that interact with the GA receptor are denoted with dotted lines. The DELLA and GRAS domains are indicated with light and dark gray arrows, respectively. Identical and similar amino acids are shaded in black and gray, respectively. (**B**) A phylogenetic tree of VvDELLA with five *Arabidopsis* DELLAs and one SlDELLA. Comparison of the Poly S/T/N motifs (**C**) between VvDELLA with SlDELLA and (**D**) between VvGAI1 with AtGAI.
(PDF)

Table S1 TIGR, SGN, or GenBank accession numbers of the proteins used for the phylogenetic analysis.
(DOCX)

Table S2 Primers for the qRT-PCR used in this study.
(DOCX)
