# Supplementary figures and images for: Gibberellin Application at Pre-Bloom in Grapevines Down-Regulates the Expressions of VvIAA9 and VvARF7, Negative Regulators of Fruit Set Initiation, during Parthenocarpic Fruit Development
Source: PLoS One. 2014 Apr 17;9(4):e95634. doi: 10.1371/journal.pone.0095634 (PMC3990702; doi:10.1371/journal.pone.0095634)

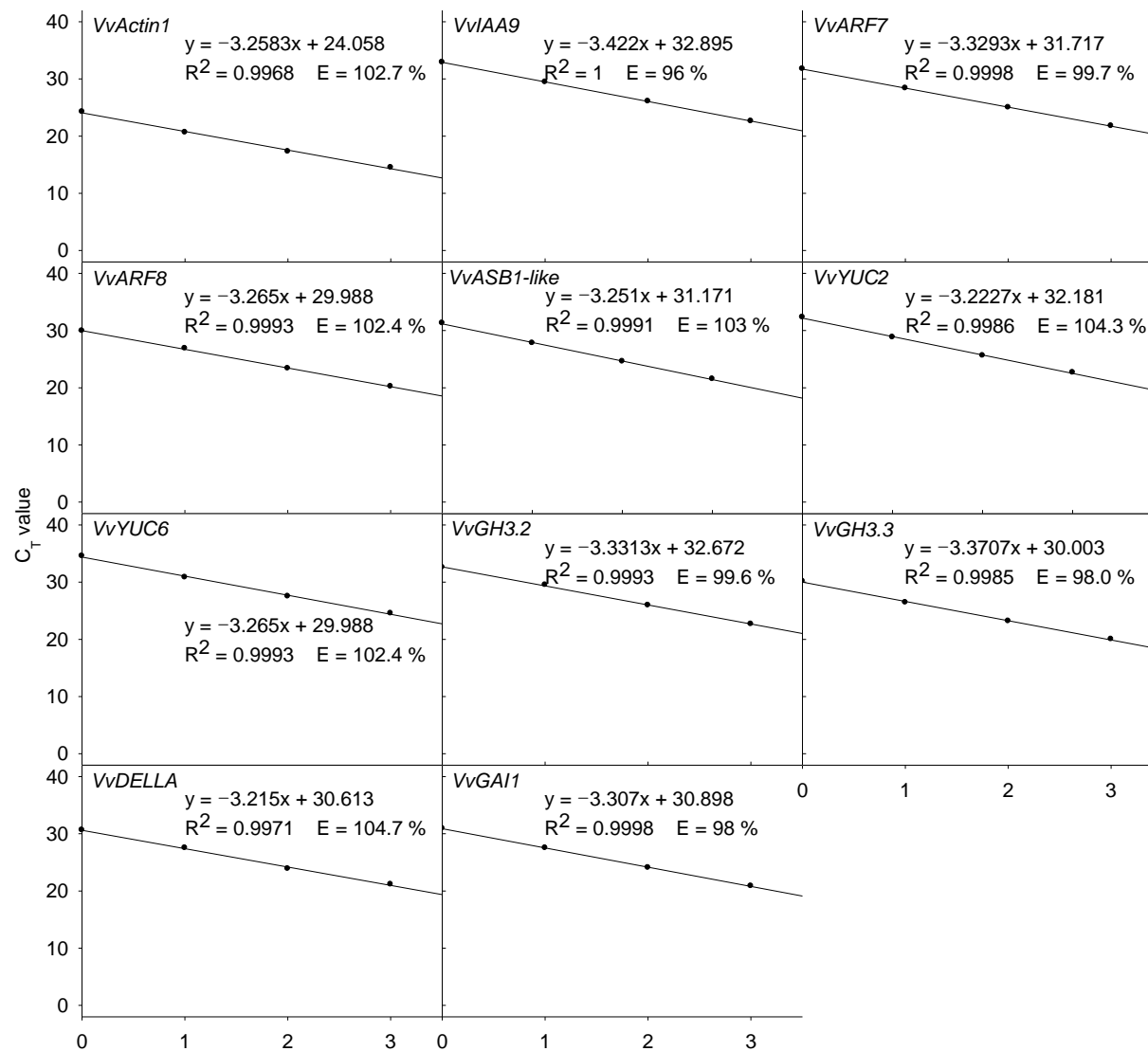

Supplement: Figure S1 — qRT-PCR efficiency plots for GA metabolic genes. Mean quantification cycle (CT) values obtained from 10-fold serial dilution series of each gene plotted against the logarithm of the cDNA template concentration. The amplification efficiency (E) was calculated by E = [10(−1/S) −1]×100, where S = the slope of the linear regression line. (PDF) [file pone.0095634.s001.pdf]
