# Supplementary material for: Gibberellin Application at Pre-Bloom in Grapevines Down-Regulates the Expressions of VvIAA9 and VvARF7, Negative Regulators of Fruit Set Initiation, during Parthenocarpic Fruit Development
Source: PLoS One. 2014 Apr 17;9(4):e95634. doi: 10.1371/journal.pone.0095634 (PMC3990702; doi:10.1371/journal.pone.0095634)
Supplement: Figure S2 — Protein alignment and phylogenetic analysis of VvARF7 and VvARF8. (A) Comparison of the AtARF7, SlARF7, and VvARF7 amino acid sequences. (B) Comparison of the AtARF8, SlARF8, and VvARF8 amino acid sequences. The B3 DNA-binding domain is denoted with an open box. The Aux/IAA dimerization domains III and IV are underlined with solid and dashed lines, respectively. Identical and similar amino acids are shaded in black and gray, respectively. (PDF) [file pone.0095634.s002.pdf]

A

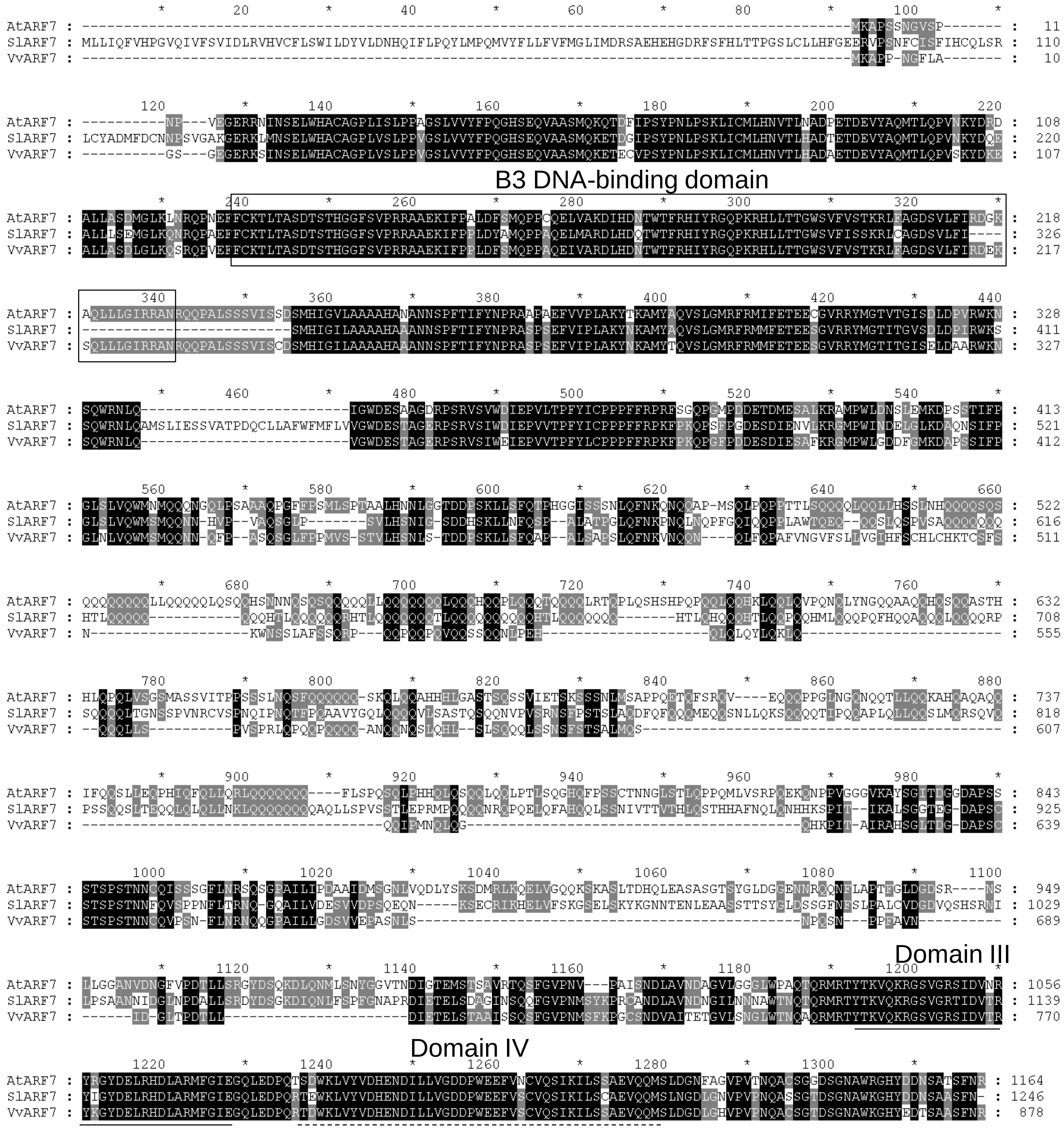

AtARF7
SlARF7
VvARF7
B3 DNA-binding domain
Domain III
Domain IV

B

```
              *        20         *        40         *        60         *        80         *
AtARF8 : MKLSTSGLG-QQGHEGE-KCLNSELWHACAGPLVSLPSSGSRVVYFPQGHSEQVAATTNKEVDGHIPNYPSLPPQLICQLHNVTMHADVET :  89
SlARF8 : MKLSTSGMG-QQAHEGENKCLNSELWHACAGPLVCLPTVGSRVVYFPQGHSEQVAATTNKELDIHIPNYPNLPPQLICPLHNVTMHADVET :  90
VvARF8 : MKLSTSGLGQQQGHEGEKKCLNSELWHACAGPLVSLPTVGSRVVYFPQGHSEQVAATTNKEVDGHIPNYPSLPPQLICQLHNVTMHADVET :  91

                                                          B3 DNA-binding domain
            100         *       120         *       140         *       160         *       180
AtARF8 : DEVYAQMTLQPLTPEEQKETFVPIELGIPSKQPSNYFCKTLTASDTSTHGGFSVPRRAAEKVFPPLDYTLQPPAQELIARDLHDVEWKFRH : 180
SlARF8 : DEVYAQMTLQPLTLQEQKDTYLPVELGIPSRQPTNYFCKTLTASDTSTHGGFSVPRRAAEKVFPPLDFSQTPPCQELIARDLHDIEWKFRH : 181
VvARF8 : DEVYAQMTLQPLTPQEQKDTFLPVELGIPSKQPTNYFCKTLTASDTSTHGGFSVPRRAAEKVFPPLDFSQQPPAQELIARDLHDVEWKFRH : 182

              *       200         *       220         *       240         *       260         *
AtARF8 : IFRGQPKRHLLTTGWSVFVSAKRLVAGDSVIFIRNEKNQLFLGIRHATRPQTIVPSSVLSSDSMHIGLLAAAAHASATNSCFTVFFHPRAS : 271
SlARF8 : IFRGQPKRHLLTTGWSVFVSAKRLVAGDSVLFIWNEKNQLFLGIRRATRPQTVMPSSVLSSDSMHIGLLAAAAHAASTNSCFIVFFNPRAS : 272
VvARF8 : IFRGQPKRHLLTTGWSVFVSAKRLVAGDSVLFIWNEKNQLLLGIRRATRPQTVMPSSVLSSDSMHIGLLAAAAHAAATNSCFTIFYNPRAS : 273

          280         *       300         *       320         *       340         *       360
AtARF8 : QSEFVIQLSKYIKAVFHTRISVGMRFRMLFETEESSVRRYMGTITGISDLDSVRWPNSHWRSVKVGWDESTAGERQPRVSLWEIEPLTTFP : 362
SlARF8 : PSEFVIPLSKYIKAVYHTRVSVGMRFRMLFETEESSVRRYMGTITGIGDLDPVRWANSHWRSVKVGWDESTAGERQPRVSLWEIEPLTTFP : 363
VvARF8 : PSEFVIPLSKYVKAVFHTRVSVGMRFRMLFETEESSVRRYMGTITGISDLDPVRWPNSHWRSVKVGWDESTAGERQPRVSLWEIEPLTTFP : 364

             *       380         *       400         *       420         *       440         *
AtARF8 : MYPSLFPLRLKRPWHAGTSSLPDGRGDLGSGLTWLRGGGGEQQGLLPLNYPSVGLFPWMQQRLDLSQMGTDNNQQYQAMLAAGLQNIGGGD : 453
SlARF8 : MYPSLFPLRLKRPFYQGTSSYQDSNNEAINRMSWLRGNAGEL-GHHSMNLQSFGMLPWMQQRVDSTILPNDINQHYQAMLATGLQSFGSGD : 453
VvARF8 : MYPSLFPLRLKRPWHPGASSLHDSRDEAANGLMWLRGETGDQ-GLQSLNFQTVGMFPWTQQRLDPTFLGNDHNQQYQAMLAAGLQNLGSGD : 454

          460         *       480         *       500         *       520         *       540
AtARF8 : PLRQQFVQLQEPHHQYLQQSASHNSDLMLQQQQQQASRHLMHAQTQIMSENLPQQNMRQEVSNQPAGQQQQLQQPDQNAYLNAFKMQNGH : 544
SlARF8 : LLKQQLMQFQQP-VQYLQHASTE?--FNFASAAAAAANNAASSSSAYAACSNPNAVREPSKAIPAQSNNQSEEQAHQHTYQEAFQLPHDQ : 540
VvARF8 : PLKQQYMQFQQP-FQYLQQTGSNN--PLLQQRQQPQVIQQTIPQHMSHAQTQILQDN-LPRHLLQQQLNNQQEQPQQQHSYQESFQIQSDQ : 541

           *       560         *       580         *       600         *       620         *
AtARF8 : LQQWQQQSEMPSPSFMKSDFTDSSNKFATTASPAS-----------GDGNLLNFSI-TGQSVLPEQLTTEGWSPKAS----NTFSEPLSLP : 619
SlARF8 : LQQRQP-SNVTSP-FLKADFADLTSKFSASVAPSGVQNMLGSLCSEGSNNSLNINR-TGQSVIIEQSPQQSWMSKFTESQLNTCSNSSSLP : 628
VvARF8 : LQQRPQ-PNVPSLSFSKADFPDSNTKFSS-ITPSSMQNMLGSMCPEGSGNLLNFSRTTGQSMLSEQPPQQPWATKFTHSQFNAFANSTSLP : 630

         640         *       660         *       680         *       700         *       720
AtARF8 : QAYPGKSLALEPG----NPQNPSLFGVDPDS-GLFLPSTVPRFASSSGDAEASPMSLTDSGFQNSLYSCMQDTTHELLHGAGQINSSNQTK : 705
SlARF8 : TY--GKDTFNPRGNCSLDSQNQSLFGANVDSSGLLLPTTVSNVATTSIDADISSMPLGTSGFPNPLYSYVQDST-DLLHNVGQADAQTVPR : 716
VvARF8 : PFT-GKDAAVEPENCNLDAQNHTLFGVNIDSSGLLLPTTVPSFGSSSVDADVSSMPLGASGFQGSLFGCVQDPS-ELLQNAGQVDPPTPSR : 719

                       Domain III                                         Domain IV
          *       740         *       760         *       780         *       800         *      82
AtARF8 : NFVKVYKSGSVGRSLDISRFSSYHELREELGKMFAIEGLLEDPLRSGWQLVFVDKENDILLLGDDPWESFVNNVWYIKILSPEDVHQMGDH : 796
SlARF8 : TFVKVYKSASLGRSLDITRFNSYHELRQELGQMFGIEGLLEDPQRSGWQLVFVDRENDVLLLGDDPWEEFVNNVWYIKILSPEDVQKLGKE : 807
VvARF8 : TFVKVYKSGSVGRSLDITRFSSYHELREELGQMFGIEGKLENPLRSGWQLVFVDRENDVLLLGDDPWEAFVNNVWYIKILSPEDVQKMGKQ : 810

         0         *       840         *       860         *       880
AtARF8 : GEGS----------------------GGLFPQNPTHL-------------------------- : 811
SlARF8 : EVGS-LNRGPPERMSSNNSADGRDFMSGLPSIGSIDY?TAVVVR?TKCGSASQELGVLLLSPN : 867
VvARF8 : GIESGFSPNSAQRMNSSGTDD-RDLVSGLPSAGSIEY-------------------------- : 846
```
