# Supplementary material for: Gibberellin Application at Pre-Bloom in Grapevines Down-Regulates the Expressions of VvIAA9 and VvARF7, Negative Regulators of Fruit Set Initiation, during Parthenocarpic Fruit Development
Source: PLoS One. 2014 Apr 17;9(4):e95634. doi: 10.1371/journal.pone.0095634 (PMC3990702; doi:10.1371/journal.pone.0095634)
Supplement: Figure S3 — Protein alignment and phylogenetic analysis of VvYUC2 and VvYUC6. (A) Comparison of the AtYUC2, AtYUC6, ToFZY2, VvYUC2, and VvYUC6 amino acid sequences. The FAD and NADPH binding sites are underlined. Identical and similar amino acids are shaded in black and gray, respectively. (B) A phylogenetic tree comparing VvYUC2 and VvYUC6 with Arabidopsis and tomato YUCCA families. (PDF) [file pone.0095634.s003.pdf]

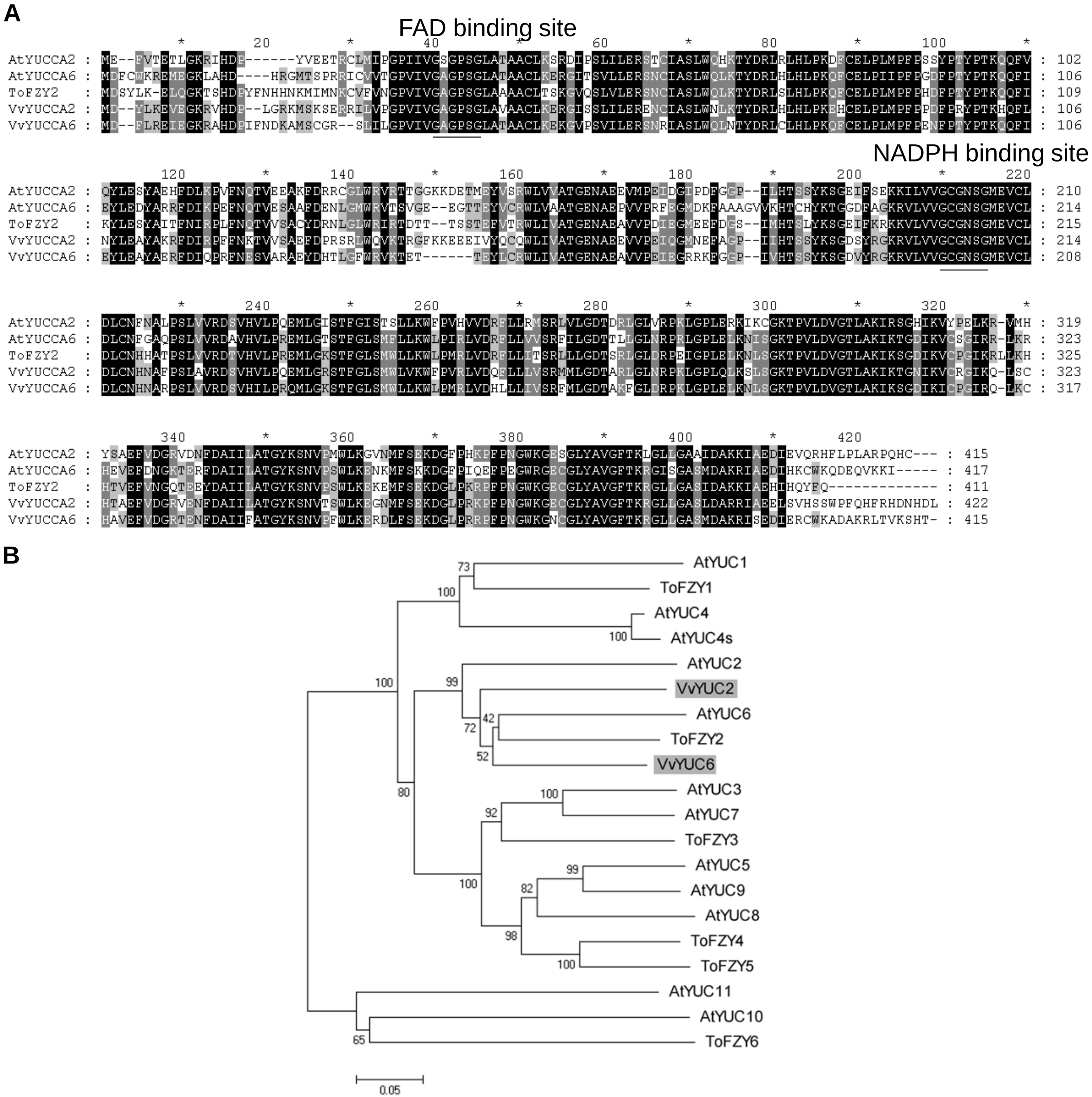
A
FAD binding site
NADPH binding site
AtYUCCA2 : ME--FVTETLGKRIHDP------YVEETRCLMIPGPIIVGSGPSGLATAACLKSRDIPSLILERSTCIASLWQHKTYDRLRLHLPKDFCELPLMPFPSSYPTYPTKQQFV : 102
AtYUCCA6 : MDFCWKREMEGKLAHD----HRGMTSPRRICVVTGPVIVGAGPSGLATAACLKERGITSVLLERSNCIASLWQLKTYDRLHLHLPKQFCELPIIPFPGDFPTYPTKQQFI : 106
ToFZY2 : MDSYLK-ELQGKTSHDFYFNHHNKMIMNKCVFVNGPVIVGAGPSGLAAAACLTSKGVQSLVLERSNCIASLWQLKTYDRLSLHLPKQFCELPLMPFPHDFPTYPTKQQFI : 109
VvYUCCA2 : MD--YLKEVEGKRVHDP--LGRKMSKSERRILVPGPVIVGAGPSGLAVAACLKERGISSLILERENCIASLWNLKTYDRLHLHLPKEHCELPLMPFPPDFPRYPTKHQFL : 106
VvYUCCA6 : MD--FLREIEGKRAHDPIFNDKAMSCGR--SLILGPVIVGAGPSGLATAACLKEKGVPSVILERSNRIASLWQLNTYDRLCLHLPKQFCELPLMPFPENFPTYPTKQQFI : 106
AtYUCCA2 : QYLESYAEHFDLKPVFNQTVEEAKFDRRCGLWRVRTTGGKKDETMEYVSRWLVVATGENAEEVMPEIDGIPDFGGP--ILHTSSYKSGEIFSEKKILVVGCGNSGMEVCL : 210
AtYUCCA6 : EYLEDYARRFDIKPEFNQTVESAAFDENLGMWRVTSVGE--EGTTEYVCRWLVAATGENAEPVVPRFEGMDKFAAAGVVKHTCHYKTGGDFAGKRVLVVGCGNSGMEVCL : 214
ToFZY2 : KYLESYAITFNIRPLFNQTVVSACYDRNLGLWRIRTDTT--TSSTEFVTRWLIVATGENAEAVVPDIEGMEEFDGS--IMHTSLYKSGEIFKRKKVLVVGCGNSGMEVCL : 215
VvYUCCA2 : NYLEAYAKRFDIRPFFNKTVVSAEFDPRSRLWQVKTRGFKKEEEIVYQCQWLIVATGENAEEVVPEIQGMNEFAGP--IIHTSSYKSGDSYRGKRVLVVGCGNSGMEVCL : 214
VvYUCCA6 : EYLEAYAERFDIQPRFNESVARAEYDHTLGFWRVKTET------TEYLCRWLIVATGENAEAVVPEIEGRRKFGGP--IVHTSSYKSGDVYRGKRVLVVGCGNSGMEVCL : 208
AtYUCCA2 : DLCNFNALPSLVVRDSVHVLPQEMLGISTFGISTSLLKWFPVHVVDRFLLRMSRLVLGDTDRLGLVRPKLGPLERKIKCGKTPVLDVGTLAKIRSGHIKVYPELKR-VMH : 319
AtYUCCA6 : DLCNFGAQPSLVVRDAVHVLPREMLGTSTFGLSMFLLKWLPIRLVDRFLLVVSRFILGDTTLLGLNRPRLGPLELKNISGKTPVLDVGTLAKIKTGDIKVCSGIRR-LKR : 323
ToFZY2 : DLCNHHATPSLVVRDTVHVLPREMLGKSTFGLSMWLLKWLPMRLVDRFLLITSRLLLGDTSRLGLDRPEIGPLELKNLSGKTPVLDVGTLAKIKSGDIKVCPGIKRLLKH : 325
VvYUCCA2 : DLCNHNAFPSLAVRDSVHVLPQEMLGRSTFGLSMWLVKWFPVRLVDQFLLLVSRMMLGDTARLGLNRPKLGPLQLKSLSGKTPVLDVGTLAKIKTGNIKVCRGIKQ-LSC : 323
VvYUCCA6 : DLCNHNARPSLVVRDSVHILPRQMLGKSTFGLSMWLLKWLPMRLVDHLLLIVSRFMLGDTAKFGLDRPKLGPLELKNLSGKTPVLDVGTLAKIKSGDIKICPGIRQ-LKC : 317
AtYUCCA2 : YSAEFVDGRVDNFDAIILATGYKSNVPMWLKGVNMFSEKDGFPHKPFPNGWKGESGLYAVGFTKLGLLGAAIDAKKIAEDIEVQRHFLPLARPQHC--- : 415
AtYUCCA6 : HEVEFDNGKTERFDAIILATGYKSNVPSWLKENKMFSKKDGFPIQEFPEGWRGECGLYAVGFTKRGISGASMDAKRIAEDIHKCWKQDEQVKKI----- : 417
ToFZY2 : HTVEFVNGQTEEYDAIILATGYKSNVPSWLKEKEMFSEKDGLPKRPFPNGWKGECGLYAVGFTKRGLLGASIDAKKIAEHIHQYFQ------------ : 411
VvYUCCA2 : HTAEFVDGRVENFDAIILATGYKSNVTSWLKEGNMFSEKDGLPRKPFPNGWKGECGLYAVGFTKRGLLGASLDARRIAEELSVHSSWPFQHFRHDNHDL : 422
VvYUCCA6 : HAVEFVDGRTENFDAIIFATGYKSNVPFWLKERDLFSEKDGLPRRPFPNGWKGNCGLYAVGFTKRGLLGASMDAKRISEDIERCWKADAKRLTVKSHT- : 415
B
AtYUC1
ToFZY1
AtYUC4
AtYUC4s
AtYUC2
VvYUC2
AtYUC6
ToFZY2
VvYUC6
AtYUC3
AtYUC7
ToFZY3
AtYUC5
AtYUC9
AtYUC8
ToFZY4
ToFZY5
AtYUC11
AtYUC10
ToFZY6
0.05
