# Supplementary material for: Gibberellin Application at Pre-Bloom in Grapevines Down-Regulates the Expressions of VvIAA9 and VvARF7, Negative Regulators of Fruit Set Initiation, during Parthenocarpic Fruit Development
Source: PLoS One. 2014 Apr 17;9(4):e95634. doi: 10.1371/journal.pone.0095634 (PMC3990702; doi:10.1371/journal.pone.0095634)
Supplement: Figure S4 — Protein sequence alignment and phylogenetic analysis of VvDELLA. (A) Comparison of the AtGAI, SlDELL, and VvDELLA amino acid sequences. The DELLA and TVHYNP motifs for GA signal perception, the poly S/T/N motif for regulation of DELLA expression and the NLS motif for nuclear localization are denoted with open boxes. The LHR and VHIID domains for dimerization of DELLA protein are indicated with solid and dashed lines, respectively. The PFYRE and SAW domains that interact with the GA receptor are denoted with dotted lines. The DELLA and GRAS domains are indicated with light and dark gray arrows, respectively. Identical and similar amino acids are shaded in black and gray, respectively. (B) A phylogenetic tree of VvDELLA with five Arabidopsis DELLAs and one SlDELLA. Comparison of the Poly S/T/N motifs (C) between VvDELLA with SlDELLA and (D) between VvGAI1 with AtGAI. (PDF) [file pone.0095634.s004.pdf]

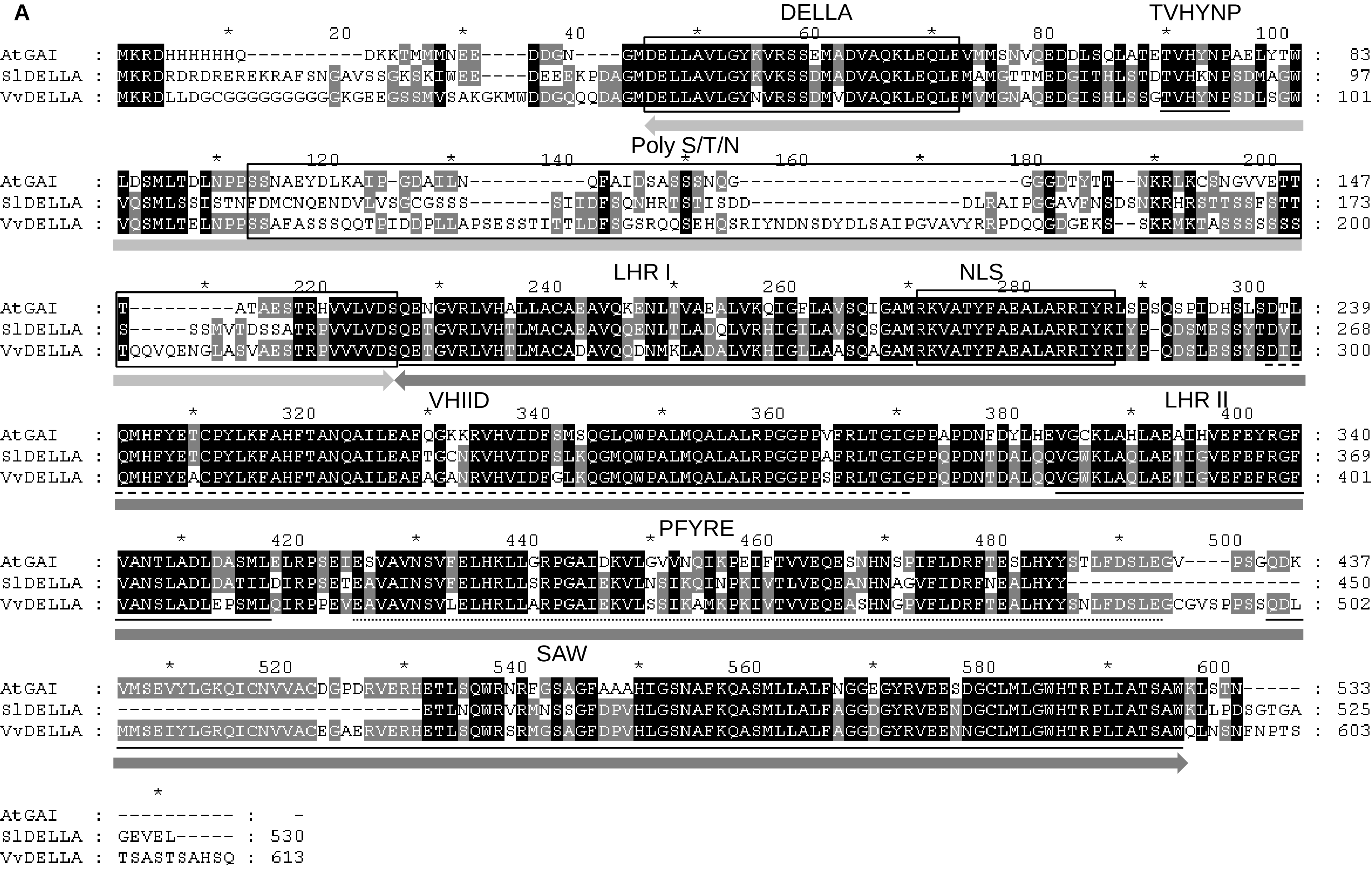
A
DELLA
TVHYNP
Poly S/T/N
LHR I
NLS
VHIID
LHR II
PFYRE
SAW
AtGAI
SlDELLA
VvDELLA

B

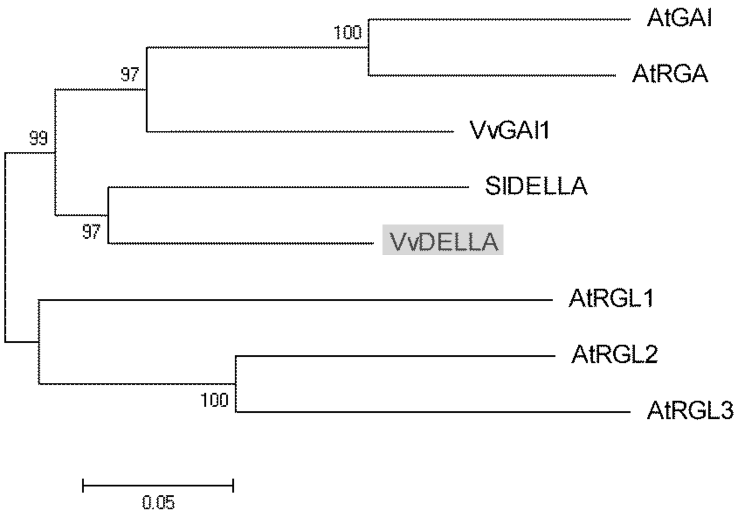

AtGAI
AtRGA
VvGAI1
SlDELLA
VvDELLA
AtRGL1
AtRGL2
AtRGL3
100
97
99
97
100
0.05

C

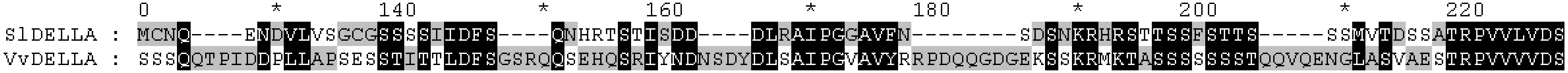

0 * 140 * 160 * 180 * 200 * 220
SlDELLA : MCNQ----ENDVLVSGCGSSSSIIDFS----QNHRTSTISDD----DLRAIPGGAVFN--------SDSNKRHRSTTSSFSTTS-----SSMVTDSSATRPVVLVDS
VvDELLA : SSSQQTPIDDPLLAPSESSTITTLDFSGSRQQSEHQSRIYNDNSDYDLSAIPGVAVYRRPDQQGDGEKSSKRMKTASSSSSSSTQQVQENGLASVAESTRPVVVVDS

D

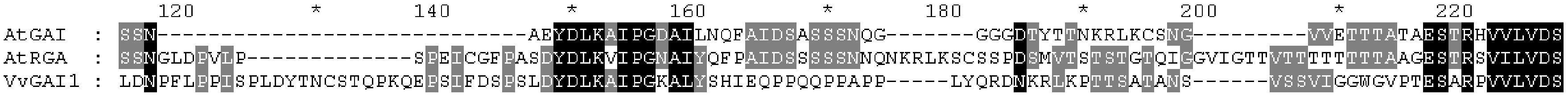

120 * 140 * 160 * 180 * 200 * 220
AtGAI : SSN-----------------------------AEYDLKAIPGDAILNQFAIDSASSSNQG-------GGGDTYTTNKRLKCSNG---------VVETTTATAESTRHVVLVDS
AtRGA : SSNGLDPVLP-------------SPEICGFPASDYDLKVIPGNAIYQFPAIDSSSSSNNQNKRLKSCSSPDSMVTSTSTGTQIGGVIGTTVTTTTTTTTAAGESTRSVILVDS
VvGAI1 : LDNPFLPPISPLDYTNCSTQPKQEPSIFDSPSLDYDLKAIPGKALYSHIEQPPQQPPAPP-----LYQRDNKRLKPTTSATANS------VSSVIGGWGVPTESARPVVLVDS
