# Supplementary material for: Gibberellin Application at Pre-Bloom in Grapevines Down-Regulates the Expressions of VvIAA9 and VvARF7, Negative Regulators of Fruit Set Initiation, during Parthenocarpic Fruit Development
Source: PLoS One. 2014 Apr 17;9(4):e95634. doi: 10.1371/journal.pone.0095634 (PMC3990702; doi:10.1371/journal.pone.0095634)
Supplement: Table S1 — TIGR, SGN, or GenBank accession numbers of the proteins used for the phylogenetic analysis. (DOCX) [file pone.0095634.s005.docx]

**Table S1.** **TIGR, SGN, or GenBank accession numbers of the proteins used for the phylogenetic analysis.**

| Protein family | Species | Protein name | Accession no. |
| --- | --- | --- | --- |
| ARF | *Arabidopsis thaliana* | AtARF1 | NP_176184 |
|  |  | AtARF2 | NP_851244 |
|  |  | AtARF3 | NP_180942 |
|  |  | AtARF4 | NP_200853 |
|  |  | AtARF5 | NP_173414 |
|  |  | AtARF6 | NP_174323 |
|  |  | AtARF7 | NP_851047 |
|  |  | AtARF8 | NP_198518 |
|  |  | AtARF9 | NP_194129 |
|  |  | AtARF10 | NP_180402 |
|  |  | AtARF11 | NP_182176 |
|  |  | AtARF12 | NP_174691 |
|  |  | AtARF13 | NP_174679 |
|  |  | AtARF14 | NP_174786 |
|  |  | AtARF15 | NP_174784 |
|  |  | AtARF16 | NP_567841 |
|  |  | AtARF17 | NP_565161 |
|  |  | AtARF18 | NP_567119 |
|  |  | AtARF19 | NP_173356 |
|  |  | AtARF20 | NP_174758 |
|  |  | AtARF21 | NP_174701 |
|  |  | AtARF22 | NP_174699 |
|  | *Solanum lycopersicum* | SlARF1 | TC199727 |
|  |  | SlARF2 | SGN-340284 |
|  |  | SlARF3 | TC19246 |
|  |  | SlARF8^*^ | TC198583 |
|  |  | SlARF5 | TC198727 |
|  |  | SlARF6 | SGN-U579438 |
|  |  | SlARF7 | TC267250 |
|  |  | SlARF8 | TC214335 |
|  |  | SlARF7^*^ | EF121545 |
|  |  | SlARF10 | ACU30063 |
|  |  | SlARF11 | TC214573 |
|  |  | SlARF12 | TC193011 |
|  |  | SlARF13 | TC206384 |
|  |  | SlARF14 | TC208143 |
|  |  | SlARF15 | TC213824 |
|  |  | SlARF16 | SGN-319318 |
|  | *Vitis vinifera* | VvARF7 | CBI27770 |
|  |  | VvARF8 | XP_002266678 |
| YUC | *Arabidopsis thaliana* | AtYUC1 | NP_194980 |
|  |  | AtYUC2 | NP_193062 |
|  |  | AtYUC3 | NP_171955 |
|  |  | AtYUC4 | NP_196693 |
|  |  | AtYUC4s | NP_850808 |
|  |  | AtYUC5 | NP_199202 |
|  |  | AtYUC6 | NP_001190399 |
|  |  | AtYUC7 | NP_180881 |
|  |  | AtYUC8 | NP_194601 |
|  |  | AtYUC9 | NP_171914 |
|  |  | AtYUC10 | NP_175321 |
|  |  | AtYUC11 | NP_173564 |
|  | *Solanum lycopersicum* | ToFZY1 | NP_001234343 |
|  |  | ToFZY2 | XP_004245254 |
|  |  | ToFZY3 | XP_004247940 |
|  |  | ToFZY4 | XP_004240470 |
|  |  | ToFZY5 | XP_004242376 |
|  |  | ToFZY6 | XP_004247468 |
|  | *Vitis vinifera* | VvYUC2 | XP_002281597 |
|  |  | VvYUC6 | XP_002281015 |
| DELLA | *Arabidopsis thaliana* | AtGAI | NP_172945 |
|  |  | AtRGA | NP_178266 |
|  |  | AtRGL1 | NP_176809 |
|  |  | AtRGL2 | NP_186995 |
|  |  | AtRGL3 | NP_197251 |
|  | *Solanum lycopersicum* | SlDELLA | NP_001234365 |
|  | *Vitis vinifera* | VvDELLA | XP_002266267 |
|  |  | VvGAI1 | XP_002284648 |
|  |  |  |  |
