# Supplementary material for: Gibberellin Application at Pre-Bloom in Grapevines Down-Regulates the Expressions of VvIAA9 and VvARF7, Negative Regulators of Fruit Set Initiation, during Parthenocarpic Fruit Development
Source: PLoS One. 2014 Apr 17;9(4):e95634. doi: 10.1371/journal.pone.0095634 (PMC3990702; doi:10.1371/journal.pone.0095634)
Supplement: Table S2 — Primers for the qRT-PCR used in this study. (DOCX) [file pone.0095634.s006.docx]

**Table S2. Primers used for the qRT-PCR used in this study.**

| Gene | Forward primer (5’ to 3’) | Reverse primer (5’ to 3’) |
| --- | --- | --- |
| *VvARF7* | CGTGCCTGTCCCAAATCAAGCT | CATCGATTAAATGAGGCAGCTGAGG |
| *VvARF8* | GGGGATCCTCTGAAACAGCAATACAT | AATGATTAATGGATTATTGCTGCCCG |
| *VvIAA9* | GGTGCCCAAACCGGTGAGTTAC | GAGCAAAAAAATTTATAACTCCAAACCTTCT |
| *VvYUC2* | CCCGGAGAATTTCCCGACTTACC | CCTTGGCTGTATATCGAACCGCTC |
| *VvYUC6* | GTGAGCGCGGAATTCGATCCT | TCCTCCTTTTTGAATCCCCGAGTC |
| *VvDELLA* | TCCCTCCTCTGCTTTCGCTTCC | GAGGACTCGGAGGGAGCAAGGA |
| *VvGAI1* | CACTCCCAATTGCGCCCTTG | CTGCAATTGGTGTAATCAAGAGGGG |
| *VvActin1* | CCCTCCTCTGCTTTCGCTTCC | GAGGACTCGGAGGGAGCAAGGA |
